# Molecular Determinants of Selectivity in Disordered Complexes May Shed Light on Specificity in Protein Condensates

**DOI:** 10.3390/biom12010092

**Published:** 2022-01-06

**Authors:** Alexander Miguel Monzon, Damiano Piovesan, Monika Fuxreiter

**Affiliations:** 1 Department of Biomedical Sciences, University of Padova, 35131 Padova, Italy; monzon.alexander@gmail.com (A.M.M.); damiano.piovesan@gmail.com (D.P.); 2 Department of Biochemistry and Molecular Biology, University of Debrecen, 4032 Debrecen, Hungary

**Keywords:** biomolecular condensates, protein interactions, disordered complexes, fuzzy interactions

## Abstract

Biomolecular condensates challenge the classical concepts of molecular recognition. The variable composition and heterogeneous conformations of liquid-like protein droplets are bottlenecks for high-resolution structural studies. To obtain atomistic insights into the organization of these assemblies, here we have characterized the conformational ensembles of specific disordered complexes, including those of droplet-driving proteins. First, we found that these specific complexes exhibit a high degree of conformational heterogeneity. Second, we found that residues forming contacts at the interface also sample many conformations. Third, we found that different patterns of contacting residues form the specific interface. In addition, we observed a wide range of sequence motifs mediating disordered interactions, including charged, hydrophobic and polar contacts. These results demonstrate that selective recognition can be realized by variable patterns of weakly defined interaction motifs in many different binding configurations. We propose that these principles also play roles in determining the selectivity of biomolecular condensates.

## 1. Introduction

Biomolecular condensates formed by liquid–liquid phase separation contribute to a wide range of cellular functions [1,2]. The droplet state is a fundamental state that can be sampled by any proteins under appropriate cellular conditions [3]. Proteins may drive the formation of condensates by spontaneously undergoing liquid–liquid phase separation or serve as droplet-clients, which can be partitioned into condensates upon post-translational modifications, interactions with nucleic acids in particular RNA [4], metabolites, altered ion concentration or pH. The generic nature of this phenomenon, however, raises questions regarding the interactions that drive the formation and stabilize the droplets. There are two major contradictory requirements. First, owing to the generality of the phenomenon, we need to assume that ‘generic’ binding motifs operate [3]. Second, forming condensates with distinguished compositions requires specific interaction motifs [5,6].

Molecular recognition is an association of selected partners through dedicated non-covalent interactions. This process requires a distinguished set of residues that form specific contacts with the partner, which can discriminate it from non-specific molecules. By ‘specific’, we mean a well-defined contact that is established between selected residues positioned in a well-defined interface. Condensates violate these requirements: First, droplet-driving regions tend to be disordered [7], in contrast to the well-defined structures in specific complexes. Second, interaction motifs encoded by low-complexity protein sequences are often redundant [8], in contrast to the distinguished interaction sites in specific complexes. Third, dynamic assembly/disassembly of condensates and variable compositions may require context-dependent interaction motifs [9,10], in contrast to the non-variable interaction motifs in specific complexes.

**Figure 1:**
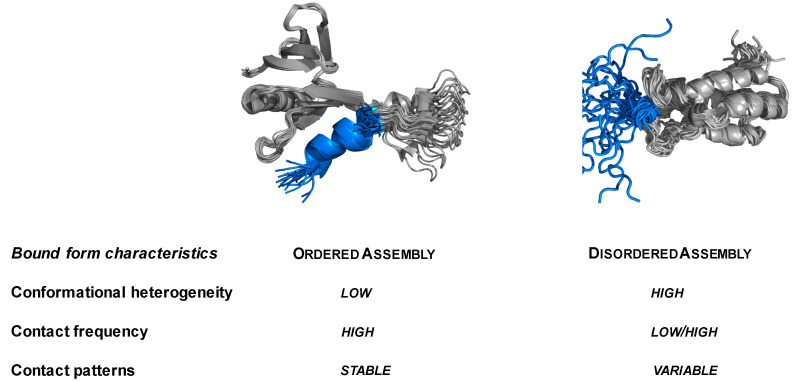
Characteristics of ordered and disordered assemblies. The ordered/disordered nature of a protein complex can be evaluated based on conformational heterogeneity and contact pattern variability. The frequency of contacts can be high in both cases. The ordered complex is represented by the amino-terminal transactivation domain (TAD) of the tumor suppressor p53; p53 (residues 47–55, blue) specifically binds pleckstrin homology (PH) domain (gray) of the Tfb1 subunit of TFIIH (PDB:2gs0 [16]). Upon binding, the disordered transactivator region of p53 forms an amphipathic alpha helix (blue). The disordered complex is represented by binding of the p53 disordered region following the oligomerisation domain (367–386 residues, blue) to the bromo domain (gray) of the transcriptional coactivator CBP (CREB binding protein). However, in the complex structure (PDB:1jsp [17]) the p53 region partly folds, it does not adopt a stable structure; instead, partly remains disordered in the bound state.

Increasing experimental evidence shows that proteins may sample a wide range of binding modes in their stoichiometric complexes, from ordering to disordering upon binding coupled to decrease or increase of conformational entropy [11,12]. Disordered binding modes observed in specific assemblies of both structured and disordered proteins are achieved via many different binding configurations, leading to conformational heterogeneity in the bound state (Figure 1) [13]. Protein sequences capable of forming disordered interactions lack the local compositional bias for ordered binding, and this feature is, in particular, enriched in sequences of droplet-driving proteins [14]. Due to the sensitivity of protein interactions to the cellular context, many protein regions can sample both disordered and ordered binding modes [15].

Disordered regions, which drive phase separation, often form disordered or fuzzy complexes with their specific partners [18]. In the absence of high-resolution structural information on condensates, however, the molecular details of the disordered interactions remained to be elucidated. The GCN4 transactivator region (1–134 residues), for example, is essential for condensation of the transcription factor; at the same time, this region is engaged in a fuzzy complex with the Med15 co-activator [19]. The interface, which cannot be described via a single conformation, is formed via various hydrophobic residues, which interact with residues in the shallow hydrophobic cleft of Med15. Nuclear magnetic resonance (NMR) studies rationalized mutation ambiguity, which originates in the interaction ambiguity at the interface. Furthermore, structure–function studies have demonstrated that transcriptional activity is compromised by stabilizing the helix position and reducing conformational heterogeneity [20]. The partner Med15 is also capable of forming biomolecular condensates by using the same region that interacts with GCN4 [21]. These studies suggest that stoichiometric and non-stoichiometric disordered assemblies, such as those in protein condensates, may be stabilized by similar interactions.

Here we analyze the structural and interaction properties in specific disordered complexes of proteins, including those which were observed to drive formation of protein droplets [22]. Our study provides a characterization of the molecular determinants of selectivity in this mode of recognition [23]. We find conformational heterogeneity and ambiguous contact patterns in the interfaces of these specific complexes, independently on the static or dynamic nature of the contacts. We demonstrate that specific recognition can be achieved via a combination of a wide range of ordered and disordered interacting residues, while lacking distinguished binding motifs. We propose that these organizing principles of stoichiometric protein assemblies also operate in higher-order assemblies, such as biomolecular condensates.

## 2. Results

### 2.1. Specific Complexes of Droplet-Driver Proteins Are Conformationally Heterogeneous

Droplet-driver proteins were assembled from public resources, primarily the PhaSepDB database (http://db.phasep.pro/ [22] (accessed on July 2021), and previously compiled datasets [18] based on experimental evidence that a protein is capable to spontaneously undergo liquid–liquid phase separation (D*_EXP_*; Supplementary Materials Table S1). As phase-separating proteins are discovered with a rapid pace, and likely more proteins are capable of driving phase separation than deposited in public datasets, we have also assembled complexes of proteins with high probability to undergo liquid–liquid phase separation, using the FuzDrop method [18] (D*_PRED_*; Supplementary Materials Table S1). Then we searched for structures of all specific complexes of these proteins, which were determined by NMR methods (Supplementary Materials Table S1; Methods). These datasets represented stoichiometric, specific complexes of condensate-driver proteins based on experimental evidence or predictions. Protein regions in these complexes may represent the region, which is critical for condensate-formation (for example GCN4 [21], see other examples in FuzDB, fuzdb.org [13]; Supplementary Materials Table S1). In most cases, however, the droplet-driving regions are not well-defined. In addition to complexes of droplet-driver proteins, we have assembled a dataset of specific disordered assemblies (SDA), formed by proteins, which are not known to form condensates (Supplementary Materials Table S1; Methods).

**Figure 2:**
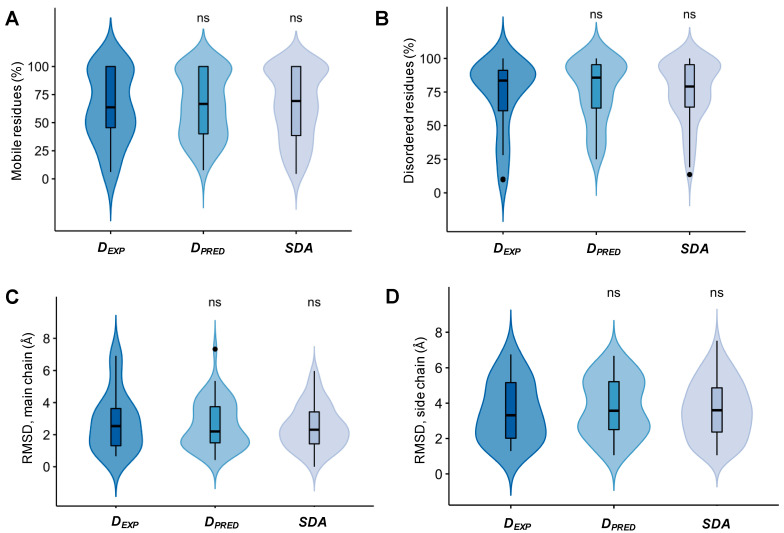
Conformational heterogeneity in specific complexes of experimental droplet-driving proteins (D*_EXP_*), predicted droplet-driving proteins (D*_PRED_*) and specific disordered assemblies (SDA). (**A**) The percentage of mobile residues in the disordered protein based on the Mobi method [24]. (**B**) The percentage of disordered residues by the DSSP method [25] based on secondary structure classifications. (**C**) Root-mean-square deviations (RMSD) of the main chain residues. (**D**) Root-mean-square deviations (RMSD) of the side chain residues. Experimental (D*_EXP_*) and predicted droplet-driving proteins (D*_PRED_*) and specific disordered assemblies (SDA) exhibit similar degrees of conformational heterogeneity based on these four properties. Statistical significances were computed by the Mann–Whitney test, using the R program (ns: *p* > 0.05).

We have analyzed the structural and interface properties of specific assemblies of experimental and predicted droplet-forming proteins and compared them to other disordered assemblies. First, we computed what fraction of residues of the disordered partner remained disordered in the bound assembly. Based on the analysis of all deposited models, we found that over 60% of the residues can be classified as mobile by the Mobi method [24], based on local conformational parameters (Figure 2A). Furthermore, over 75% of the residues lack a regular secondary structure based on the DSSP classification [25] (Figure 2B). Datasets of disordered protein complexes, components of which were not known to form droplets (Methods and Supplementary Materials Table S1) exhibit a similar degree of structural disorder as that of droplet-forming proteins (Figure 2A,B). In addition, we have computed the root-mean-square deviation (RMSD) between the different models in each ensemble (Methods section). We found a considerable deviation between main chain (RMSD ~2.5 Å) and side chain (~4.0 Å) atoms in all datasets (Figure 2C), suggesting that proteins may sample completely different conformations when bound with their specific partners. Taken together, these results indicate a large degree of conformational heterogeneity in specific disordered protein assemblies.

### 2.2. Droplet-Forming Proteins Sample Multiple Binding Configurations in Specific Assemblies

**Figure 3:**
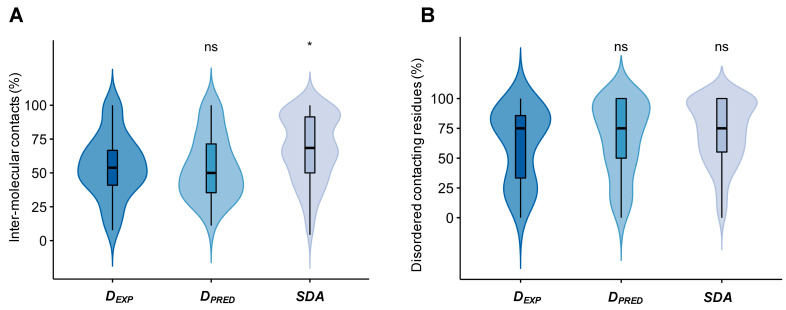
Residues involved in persistent contacts (f_C_ ≥ 0.75; f_C_ = N_C_/N_TOTAL_, where N_C_ is the number of models when the residue is in contact, and N_TOTAL_ is the number of all models) in specific complexes of experimental droplet-driving proteins (D*_EXP_*), predicted droplet-driving proteins (D*_PRED_*) and specific disordered assemblies (SDA). (**A**) The percentage of interface residues that are involved in partner contacts in at least three-quarters of the conformers of the ensemble. (**B**) The percentage of disordered residues in the pool of residues with persistent contacts (f_C_ ≥ 0.75); based on the DSSP method. Statistical significances were computed by the Mann–Whitney test, using the R program (ns: *p* > 0.05, * *p* ≤ 0.05).

Then we analyzed whether residues that form specific contacts with the partner are also conformationally heterogeneous. First, we determined whether the interface residues, which are involved in inter-chain contacts in at least one model, formed stable or transient interactions with the partner. To this aim, we classified residues according to whether they were in contact with the partner in the given model of the ensemble (<4.5 Å separation; Methods section) and then computed the fraction of models, where contacts with any partner residue were observed (f_C_ = N_C_/N_TOTAL_, where N_C_ is the number of models when the residue is in contact, and N_TOTAL_ is the number of all models; Methods section). Despite the large variation in conformation, we found that most residues were in contact with the partner in the different models. In total, 50–57% of the residues in droplet-forming proteins were in contact with the partner (f_C_ ≥ 0.75; at least in three quarters of the conformers), and in case of specific disordered assemblies, this ratio was close to 70% (Figure 3A). Only about one-third of the droplet-forming protein residues were in contact in less than one-quarter of the models. This indicates that, despite the conformational heterogeneity of the assemblies, most residues could mediate interactions with the partner.

Then we analyzed the structural properties of the contacting residues. We found that 75% of those residues that formed permanent interactions (f_C_ ≥ 0.75) with the partner were disordered by the DSSP method (Figure 3B). Both main chain (RMSD ~1.9 Å), and side-chain (~2.9 Å) atoms of these residues exhibited large structural variability, while in contact with the partner (Table 1). The structural deviations of residues that established more transient contacts (f_C_ ≤ 0.25) were even larger (Table 1). Taken together, these data indicate that, in their specific assemblies, contacting residues of droplet-forming proteins sample multiple binding conformations.

### 2.3. Different Contact Patterns Mediate Specific Interactions in Disordered Assemblies

**Figure 4:**
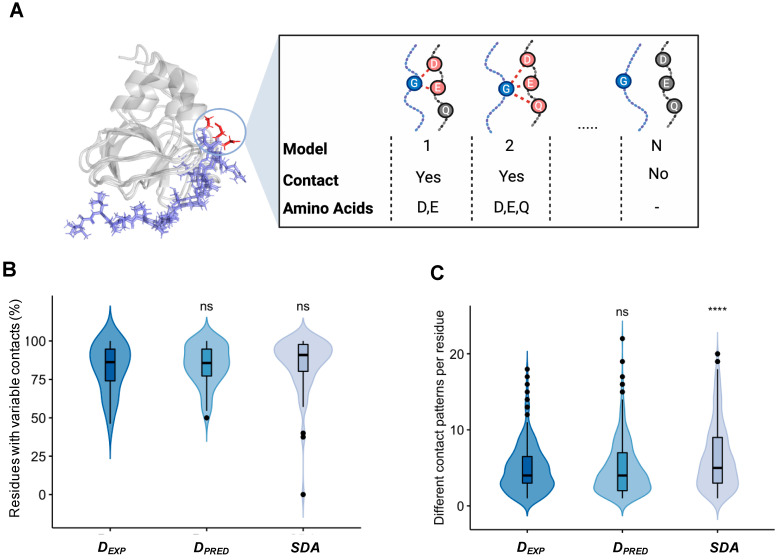
Contact-pattern variability in specific complexes of experimental droplet-driving proteins (D*_EXP_*), predicted droplet-driving proteins (D*_PRED_*) and specific disordered assemblies (SDA). (**A**) Definition of different contact patterns, as illustrated by the complex of Myc proto-oncogene protein (blue) with Myc box–dependent interacting protein 1 (gray) (PDB:1mv0). Contacting residues (in any of the conformers) are shown in red. (**B**) Percentage of residues with changing contact patterns in the ensemble. (**C**) Frequency of the different contact patterns within the conformational ensembles of the three groups of disordered assemblies. The disordered assemblies of experimental droplet-driving proteins (D*_EXP_*), predicted droplet-driving proteins (D*_PRED_*) and specific disordered assemblies (SDA) exhibit similar degrees of interaction variability. Specific disordered assemblies of those proteins, which are not known to phase separate, exhibit more contact patterns than those of droplet-driving proteins. Statistical significances were computed by the Mann–Whitney test, using the R program (ns: *p* > 0.05, **** *p* ≤ 0.0001).

Then we asked the question of how selectivity is achieved with conformationally variable contact residues. Thus, we determined the interaction pattern at the interface, defined as the pairs of all contacting residues in each conformer of the ensemble (Methods section). Then we analyzed whether the contacts of a given residue were identical in the different models of the ensemble and computed the frequency of each contact pattern (Figure 4A; Methods section). Here we did not apply any constrains on the number of the residue contacts. Surprisingly, more than half of the contact residues in experimental and in predicted droplet-forming proteins interacted with different sets of residues on the partner (Figure 4B), similarly to specific disordered assemblies (Figure 4B). In addition, we determined the number of different contact patterns per residue (Figure 4C; Methods section), which also demonstrated large contact variability in specific complexes. These results indicate that ambiguous interactions can play roles in specific recognition of selected targets.

**Figure 5:**
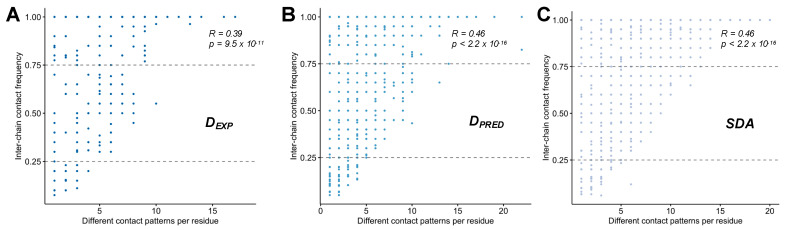
Relationship between contact persistence and variability in (**A**) experimental droplet-driving proteins (D*_EXP_*), (**B**) predicted droplet-driving proteins (D*_PRED_*) and (**C**) specific disordered assemblies (SDA). The extent of contact-pattern variation (shown on the *x*-axis) does not correlate with the frequency of inter-chain contacts (*y*-axis). Contact-pattern variations were defined as shown in Figure 4, and contact frequency was computed as f_C_ = N_C_/N_TOTAL_, where N_C_ is the number of models when the residue is in contact, and N_TOTAL_ is the number of all models.

Then we asked whether changes in contact patterns are associated with more transient interactions. Thus, we analyzed the relationship between variability of contact patterns and frequency of these contacts. We found that overall the variability of contact patterns weakly correlates with the frequency, i.e., static or dynamic nature of the contacts (Figure 5). These results corroborate that multiple binding modes may involve different sets of interactions between the selected partners.

### 2.4. Specific Interactions Can Be Realized via a Wide Range of Sequence Motifs

**Figure 6:**
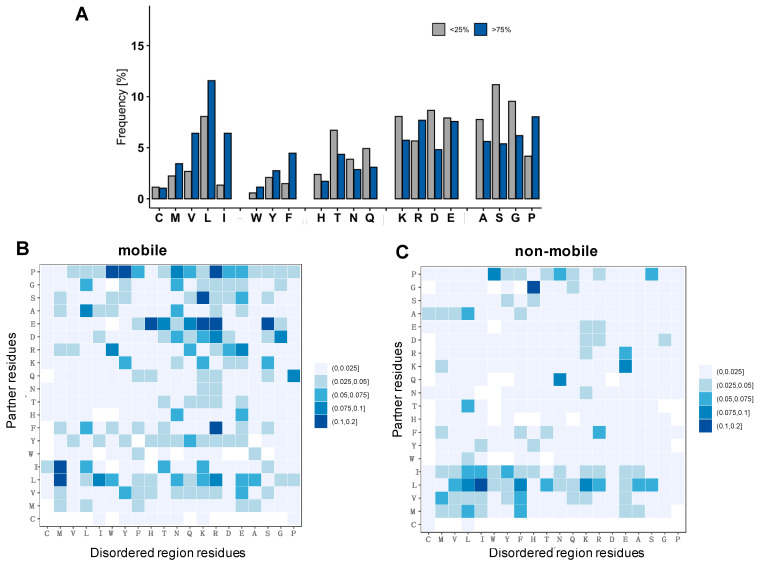
Compositional analysis of interfaces of droplet-driving proteins. (**A**) Composition of residues that establish persisting (f_C_ = N_C_/N_TOTAL_ ≥ 0.75, blue) and transient (f_C_ = N_C_/N_TOTAL_ ≤ 0.25, gray) contacts with the partner. Amino acids are grouped as hydrophobic, aromatic, polar and aggregation, promoting charged and disorder-biasing [27] residues. (**B**,**C**) Frequencies of contacting amino acids (F_ij_, Methods) in mobile (**B**) and non-mobile (**C**) residues. The *x*-axis shows the amino acids of the droplet/disordered region, and the *y*-axis shows the amino acids of the binding partner. The contacting frequency was computed by counting for the different types of contacts divided by the total number of contacts in each ensemble and taking the median value for all complexes. The color scale is shown on the right, frequencies were multiplied by 10 for visual clarity (Methods, Equation (5)). While mobile contacts are established mostly between polar and charged residues, non-mobile contacts are mostly based on hydrophobic and aromatic contacts.

Then we asked, what is the chemical nature of interactions of disordered complexes of droplet-forming proteins? First, we analyzed the composition of the interfaces and observed that residues that are involved in more stable contacts (f_C_ ≥ 0.75) are enriched in hydrophobic amino acids (Figure 6A). In contrast, residues that are involved in more transient contacts (f_C_ ≤ 0.25) are enriched in charged amino acids and those biasing for structural disorder (Figure 6A). We also compared these compositions with those of disordered regions, which are considered to drive liquid–liquid phase separation. We found, however, that interfaces of disordered protein assemblies of droplet-forming proteins differ from compositions of disordered proteins in DisProt (version 8.2) (Supplementary Materials Figure S1). In particular, we did not find enrichment of aromatic residues, which were proposed to mediate interactions in droplets [26]. This is consistent with the results, i.e., that inclusion of pi interactions did not improve the accuracy of predictions of droplet-promoting regions [18].

Then we analyzed the frequency of pair-wise contacts between the different amino acid residues (*F_ij_*, Methods Equation (5)). We found that contacts mediated by mobile, disordered residues are enriched in charge–charge interactions (Figure 6B). In interactions mediated by residues classified as non-mobile, we found more hydrophobic interactions (Figure 6C). We did not find, however, distinguished motifs dominated by any amino acids, suggesting that disordered, variable interactions may not require specific compositions; instead, they can be realized by many different combination of contacts.

## 3. Discussion and Conclusions

Biomolecular condensates formed by liquid–liquid phase separation are associated with a wide-range of biological activities [2]. Overall, the formation of protein droplets is a complex process which is determined by both intrinsic and extrinsic factors. In particular, the formation of condensates is also regulated by osmotic stress [28,29,30]. Owing to their variable stoichiometry and heterogeneous conformations, their molecular organization remains to be understood, particularly the nature of the interactions underpinning the droplets [31]. In addition to high concentration, variable stoichiometry and conformational heterogeneity, which all present bottlenecks for high-resolution structural studies of biomolecular condensates, one also needs to consider the impact of a wide range of cellular factors [32]. Stoichiometric complexes of droplet-forming proteins are characterized at high resolution [13], which can provide insights into the intrinsic factors driving liquid–liquid phase separation.

It has been demonstrated previously that droplet-formation is driven by disordered interactions [18]. Here we investigated the molecular details of such interactions, as exemplified by specific disordered assemblies of proteins. We identified three characteristic features: First, these specific complexes are conformationally heterogeneous and cannot be characterized by a unique structure. Second, residues forming contacts at the interface also sample many conformations. Third, different patterns of contacting residues are observed at the specific interface.

These observations are in line with recent results demonstrating that disorder-to-order transition of disordered regions is often, perhaps in most cases, an energetically unfavorable scenario of binding [33]. The presence of suboptimal structures leads to interaction versatility with respect to conformations and binding partners [12]. This scenario cannot be simplified as ‘dynamic’ interaction, as it is not necessarily linked to conformational exchange in the bound state. Furthermore, it is not necessarily mediated by multiple binding motifs. As we demonstrated in this study, disordered interactions can be achieved by using a wide range of sequence motifs, which lead to multiple binding configurations, even in specific assemblies of proteins.

Taken together, our results demonstrate that specific recognition can be achieved via many binding configurations, which may not require distinguished motifs. These results rationalize why biomolecular condensates exhibit weak specificity yet high configurational entropy. Future experimental work on high-resolution structural analysis of condensates or mutational studies guided by variable contact maps of specific disordered assemblies will probe this model.

## 4. Methods

### 4.1. Dataset of Disordered Protein Complexes

We searched for disordered protein assemblies in PDB, conformational ensembles of which were determined by NMR. First, protein complexes, both homomeric and heteromeric, were assembled from the Protein Data Bank (PDB) [34]. Second, the contact residues were identified in any of the models of the ensemble by using the RING software [35]. Third, these were classified as mobile and non-mobile based on the MOBI method [24]. Complexes that contained ‘mobile’ interface residues, according to the MOBI method, in any of the models of the ensemble were defined as a disordered complex (316 complexes) [24]. Then we divided this dataset based on whether the proteins involved form condensates. We used our previously compiled dataset of droplet-driving proteins [18] and the updated version of the PhaSepDB (http://db.phasep.pro/ [22] (accessed on July 2021) database, complemented with some searches of the literature to identify proteins classified as experimental droplet-drivers (*D_EXP_*; Supplementary Materials Table S1). From PhaSepDB, the category ‘PS-self’ was considered as proteins driving condensate formation. Complexes of proteins with high probability to undergo liquid–liquid phase separation based on the sequence-based FuzDrop method [18] were assembled (D*_PRED_*; Supplementary Materials Table S1). The remaining complexes of proteins that are not known to form droplets were classified as specific disordered assemblies (SDA; Supplementary Materials Table S1). Then we filtered these data to have a comparable interface length (≤50 residues) and comparable number of models in the ensemble (≤40 conformers), using only protein–protein complexes. The final dataset contained 26 complexes of 15 experimental droplet-driver proteins and 64 complexes of 51 predicted droplet-driver proteins and 101 specific disordered assemblies of 63 disordered proteins (Supplementary Materials Table S1).

### 4.2. Assessing Structural Heterogeneity in Conformational Ensembles

Root-mean-square Deviation (RMSD) was computed between all pairs of models, using the Bio.PDB.Superimposer module as implemented in BioPython, and were averaged for the ensemble. Regular secondary structures were determined with DSSP version 3.1.4, using the classifications ordered (H Alpha helix, G 3_10_ helix, I π-helix, E Strand and B beta-bridge) and disordered (T Turn, S Bend, unassigned residues). In the case over 50% of the residues were classified as disordered, the ensemble was classified as disordered based on DSSP.

### 4.3. Characterization the Variability of Inter-Chain Contacts

Two residues were defined as contacting if at least one pair of atoms in the two chains was within 4.5 Å. We analyzed the contacts of each residue and determined the fraction of models in the ensemble in which the given residue was in contact with any atoms of the partner:

f_C_ = N_C_/N_TOTAL_ (1)

where N_C_ is the number of models when the residue is in contact, N_TOTAL_ is the number of all models and f_C_ characterizes the persistence of the interactions of the given residue. In each model, we determined the contact pattern of each residue as a union of all contacting partner residues. If a residue established contacts with the same partner residues, the contact pattern remained the same. Then we determined the number of contact patterns that were observed in the ensemble for each residue (Figure 3A). The contact variability was defined as follows:

f_V_ = (N_CP_ − 1)_/_(N_C,TOT_ − 1) (2)

where N_CP_ is the number of contact patterns, and N_C,TOT_ is the number of models, where the residue was in contact models in a given ensemble. An example is if residue A interacts with two different sets of partner residues in 8 models out of 10 in the ensemble f_V_ = (2 − 1)/(8 − 1) = 0.14. With this formula, f_V_ is scaled between 0 and 1; that is, when the pattern is fixed (same on all models), f_V_ equals zero. The contact variability was computed by using in-house R scripts.

### 4.4. Composition of the Interface

The frequency of each of the 20 amino acid types was computed as follows:

AA_i_ = N_i_/N_SEQ_ (3)

where N_i_ is the number of amino acid type i, and N_SEQ_ is the length of the sequence. The compositions were determined, including all proteins in the dataset.

The properties of D*_EXP_*, D*_PRED_* and SDA were also compared with disordered regions’ composition in the DisProt database [27]. The difference in composition from the disordered regions in the DisProt database was determined as follows:

ΔAA_i_ = AA_i_ − AA_i,IDP_ (4)

where AA_i_ and AA_i,IDP_ is the frequency of amino acid type i in disordered protein complexes datasets (D*_EXP_*, D*_PRED_* and SDA) and the DisProt database.

### 4.5. Frequency of Contacting Residues

Frequencies of contacting amino acid pairs (*F_ij_*) were calculated as follows:

*F_ij_* = median {A_ij_} * (N_C_ (A_ij_)/ N_C,TOT)_ (5)

where $A_{ij}$ = $N_{ij}/\sum N_{ij}$ is the frequency that amino acids *i* and *j* are in physical contact in any of the models of the complex ($N_{ij}$) versus all possible contacts (the summation goes over all possible pairs in all the models). Then the median value, where $A_{ij}$ > 0 is computed over all the complexes {$A_{ij}$}. This is normalised by the fraction of all complexes $N_{C,TOT}$, where the given contact between amino acids *i* and *j* was observed $N_{C}\;\left(A_{ij}\right)$.

## Figures and Tables

**Table 1:** Structural variations in specific assemblies of experimentally identified (D*_EXP_*) and predicted (D*_PRED_*) droplet-forming proteins and specific disordered assemblies (SDA). Root-mean-square deviations (RMSD) are computed for residues establishing partner contacts in less than 0.25 of the models (*f_C_* ≤ 0.25) and residues forming contacts in more than 0.75 of the models (*f_C_* ≥ 0.75); *f_C_* = N_C_/N_TOTAL_, where N_C_ is the number of models when the residue is in contact, and N_TOTAL_ is the number of all models.

Datase	Main-Chain RMSD (Å)		Side-Chain RMSD (Å)	
	*f_C_* ≤ 0.25	*f_C_* ≥ 0.75	*f_C_* ≤ 0.25	*f_C_* ≥ 0.75
D*_EXP_*	2.71	1.93	3.52	2.91
D*_PRED_*	2.88	1.77	4.77	2.65
SDA	3.10	1.81	4.54	2.80

## Supplementary Materials

The following supporting information can be downloaded at: https://www.mdpi.com/article/10.3390/biom12010092/s1, Figure S1: Difference in amino acid composition of droplet-driving proteins and disordered proteins in the DisProt database. (version 8.2); Table S1: Datasets of disordered protein assemblies.
