# Supplementary material for: Molecular Determinants of Selectivity in Disordered Complexes May Shed Light on Specificity in Protein Condensates
Source: Biomolecules. 2022 Jan 6;12(1):92. doi: 10.3390/biom12010092 (PMC8773858; doi:10.3390/biom12010092)
Supplement: Supplementary file 1 [file biomolecules-12-00092-s001.zip › biomolecules-1513591-supplementary.pdf]

biomolecules

Supplementary Material

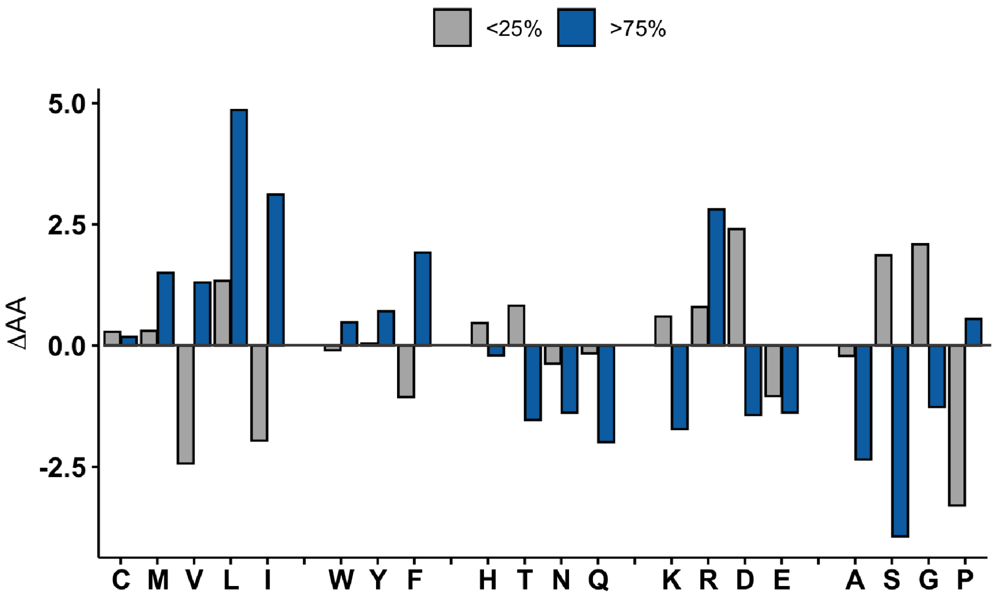

Figure S1. Difference in amino acid composition of droplet-driving proteins and disordered proteins in the DisProt database**.** (version8.2) [1]. Difference in composition is shown for residues, which establish persisting ($f_C = N_C/N_{TOTAL} \geq 0.75$, blue) and transient ($f_C = N_C/N_{TOTAL} \leq 0.25$, gray) contacts with the partner. Amino acids are grouped as hydrophobic, aromatic, polar and aggregation pro- moting, charged and disorder-promoting residues. Residues establishing persisting contacts are depleted in disorder-promoting res-idues as compared to the DisProt database.

Table S1. Datasets of disordered protein assemblies.

UniProt	Dataset	PDB Chain	Number of Models	Interface Length	FuzDB ID
O75928	D_exp	2asq_B	10	14	FC00128
P01106	D_exp	1mv0_A	20	13	FC00074
P03069	D_exp	2lpb_B	13	28	FC00061
P03069	D_exp	2g9j_A	10	20	FC00289
P03069	D_exp	2g9j_B	10	20	FC00289
P04050	D_exp	2l0i_B	10	9	
P04050	D_exp	5lvf_B	20	16	
P04050	D_exp	5m9d_B	20	16	
P04637	D_exp	2l14_B	20	38	FC00084
P04637	D_exp	1jsp_A	20	11	FC00114
P04637	D_exp	1dt7_X	40	13	
P04637	D_exp	1dt7_Y	40	13	
P04637	D_exp	1hs5_A	20	26	
P04637	D_exp	1hs5_B	20	26	
P04637	D_exp	2gs0_B	20	13	
P04637	D_exp	2ly4_B	10	39	
P04637	D_exp	2mwo_B	20	15	
P04637	D_exp	2mwp_B	20	12	

P06241	D_exp	2mrk_B	27	10	FC00122
P10636	D_exp	1i8h_A	10	13	FC00131
P12978	D_exp	2mkr_B	20	9	FC00175
P24928	D_exp	2lto_B	20	11	FC00149
P51532	D_exp	6bgh_B	20	12	FC00119
Q04206	D_exp	2lsp_A	20	13	FC00111
Q04206	D_exp	5urn_B	20	20	FC00112
Q06787	D_exp	2la5_B	10	17	FC00153
Q13094	D_exp	2ror_B	20	14	
Q6VMQ6	D_exp	2rpq_B	20	11	FC00121
Q9BZB8	D_exp	2n1o_B	19	8	FC00143
O43524	D_pred	6mnl_A	20	16	FC00164
O75496	D_pred	2lp0_B	10	18	FC00150
P02452	D_pred	2llp_A	30	16	FC00151
P02452	D_pred	2llp_B	30	16	FC00151
P02452	D_pred	2llp_C	30	16	FC00151
P04608	D_pred	6mce_B	10	17	FC00166
P04608	D_pred	6mcf_B	10	15	FC00166
P06876	D_pred	2agh_A	20	17	FC00126
P06876	D_pred	1sb0_B	20	21	
P07174	D_pred	2mic_B	10	22	FC00249
P07276	D_pred	2lox_B	20	19	FC00178
P13051	D_pred	1dpu_B	30	13	FC00190
P14737	D_pred	1fhr_P	20	7	FC00189
P14737	D_pred	1k2n_P	20	9	FC00189
P14737	D_pred	1k3n_B	20	8	FC00189
P14737	D_pred	1k3q_B	20	10	FC00189
P15381	D_pred	6ctb_B	4	19	
P16220	D_pred	2lxt_C	20	20	FC00152, FC00259
P17679	D_pred	1y0j_A	20	18	FC00296
P17679	D_pred	2l5e_B	20	6	FC00297
P19429	D_pred	1lxf_I	30	15	FC00305
P19429	D_pred	1mxl_I	40	15	FC00305
P19429	D_pred	1ozs_B	30	20	FC00305
P19429	D_pred	2kgb_I	20	18	FC00305
P19429	D_pred	2krd_I	20	16	FC00305
P19429	D_pred	2l1r_B	20	20	FC00305
P19429	D_pred	2mzp_I	20	23	FC00305
P22059	D_pred	2rr3_B	20	36	FC00169
P26675	D_pred	1aze_B	10	9	FC00324
P29474	D_pred	2mg5_B	20	16	FC00242
P29474	D_pred	2n8j_B	20	22	FC00242
P30311	D_pred	1i8g_A	10	9	FC00314
P34217	D_pred	2a0t_B	20	9	FC00186
P37088	D_pred	2m3o_P	15	11	FC00176
P37840	D_pred	2m55_B	20	18	
P42226	D_pred	5nwm_B	20	30	FC00199
P46108	D_pred	2ms4_B	20	9	FC00163
P50542	D_pred	4bxu_B	10	11	FC00168

P53632	D_pred	2mow_B	20	12	FC00124
P55036	D_pred	1uel_B	20	26	FC00154
Q00416	D_pred	6gc3_B	20	12	
Q00560	D_pred	2bbu_B	20	15	FC00293
Q01831	D_pred	2rvb_A	20	37	FC00229
Q02384	D_pred	1gbr_B	29	15	FC00317
Q03164	D_pred	2lxs_B	20	19	FC00125
Q03164	D_pred	2lxt_B	20	19	FC00125, FC00259
Q03164	D_pred	2agh_C	20	30	FC00126
Q12983	D_pred	2j5d_A	16	37	FC00183
Q12983	D_pred	2j5d_B	16	37	FC00183
Q13153	D_pred	1zsg_B	30	21	FC00129
Q13351	D_pred	2l2i_B	20	26	
Q15054	D_pred	2n1g_B	20	15	FC00172
Q15116	D_pred	6r5g_B	10	11	FC00208
Q1WCB7	D_pred	2khs_B	20	31	FC00274
Q3UND0	D_pred	1m0v_B	20	7	FC00308
Q60787	D_pred	2eu0_B	20	6	FC00185
Q77YH0	D_pred	2ihx_A	20	45	FC00284
Q79994	D_pred	484d_A	22	17	FC00225
Q8WWN8	D_pred	2lnw_B	20	9	FC00263
Q96G27	D_pred	1k5r_B	10	10	FC00311
Q96PU5	D_pred	2mpt_B	20	12	FC00174
Q96QB1	D_pred	2loz_B	10	14	FC00262
Q96QT6	D_pred	2l9s_A	20	33	FC00181
Q96RL1	D_pred	2n9e_A	20	10	FC00240
Q96T88	D_pred	5iay_B	20	13	FC00120
Q99075	D_pred	2m8s_B	18	10	FC00147
Q9BZ95	D_pred	2nd1_B	20	13	FC00141
Q9H211	D_pred	2le8_B	19	23	FC00180
Q9QZS2	D_pred	2mp2_C	10	25	FC00123, FC00146
O00418	SDA	5j8h_B	20	27	FC00167, FC00198
O24165	SDA	2g46_C	20	13	FC00290
O24165	SDA	2g46_D	20	13	FC00290
O35718	SDA	2jz3_A	20	15	FC00278
O95630	SDA	5ixf_B	10	14	FC00219
P00452	SDA	1qfn_B	20	18	FC00303
P00634	SDA	5jtm_E	20	25	FC00202
P00634	SDA	5jtm_F	20	25	FC00202
P00634	SDA	5jtm_G	20	25	FC00202
P00634	SDA	5jtm_H	20	25	FC00202
P00634	SDA	5jto_E	20	40	FC00202
P00634	SDA	5jto_F	20	40	FC00202
P00634	SDA	5jto_G	20	40	FC00202
P00634	SDA	5jto_H	20	40	FC00202
P00634	SDA	5jtp_E	20	22	FC00202
P00634	SDA	5jtp_F	20	22	FC00202
P00634	SDA	5jtp_G	20	22	FC00202
P00634	SDA	5jtp_H	20	22	FC00202

P01308	SDA	1a7f_A	20	21	
P01308	SDA	1ai0_A	10	21	
P01308	SDA	1ai0_I	10	21	
P01308	SDA	1aiy_A	10	21	
P01308	SDA	1aiy_G	10	21	
P01308	SDA	1hiq_A	10	21	
P01308	SDA	1hiq_B	10	30	
P01308	SDA	1his_A	15	21	
P01308	SDA	1his_B	15	25	
P01308	SDA	1hit_A	9	21	
P01308	SDA	1hit_B	9	23	
P01308	SDA	1hui_A	25	21	
P01308	SDA	1hui_B	25	26	
P01308	SDA	1jco_A	25	21	
P01308	SDA	1jco_B	25	27	
P01308	SDA	1mhi_A	20	21	
P01308	SDA	1mhi_B	20	30	
P01308	SDA	1mhj_A	20	21	
P01308	SDA	1sf1_A	15	20	
P01308	SDA	1vkt_A	10	20	
P01308	SDA	1xgl_A	10	20	
P01308	SDA	1xgl_B	10	26	
P01308	SDA	2h67_A	20	21	
P01308	SDA	2hh4_A	20	21	
P01308	SDA	2m2p_A	30	21	
P01308	SDA	2mpg_A	35	21	
P01308	SDA	2mpg_B	35	28	
P01308	SDA	2mpi_A	18	18	
P01308	SDA	2mpi_B	18	22	
P01308	SDA	2rn5_B	50	32	
P01308	SDA	5mwq_B	20	32	
P01317	SDA	5miz_A	10	19	FC00210
P01317	SDA	5miz_B	10	22	FC00210
P01317	SDA	6kh9_A	10	21	FC00210
P01317	SDA	6kh9_B	10	29	FC00210
P01317	SDA	6kha_A	10	21	FC00210
P01317	SDA	6kha_B	10	30	FC00210
P02710	SDA	1abt_B	4	6	FC00327
P02710	SDA	1idh_B	20	12	FC00327
P02710	SDA	1lxg_B	10	13	FC00328
P02829	SDA	2lsv_B	20	7	FC00260
P03079	SDA	1aou_P	22	10	FC00192
P04486	SDA	2k2u_B	20	20	FC00275
P04626	SDA	1mw4_B	10	9	FC00187
P04626	SDA	2l4k_B	10	10	FC00187
P05866	SDA	1etg_B	19	21	FC00318
P05866	SDA	1ull_B	7	16	FC00318
P06492	SDA	2phe_C	10	26	FC00050, FC00171
P06492	SDA	2phg_B	10	26	FC00170

P07243	SDA	1nyb_A	15	17	FC00306
P08050	SDA	2n8t_B	10	13	FC00241
P0AEY0	SDA	5jtq_E	20	42	FC00218
P0AEY0	SDA	5jtq_F	20	42	FC00218
P0AEY0	SDA	5jtr_E	20	40	FC00218
P0AEY0	SDA	5jtr_F	20	40	FC00218
P0AEY0	SDA	5jtr_G	20	40	FC00218
P0AEY0	SDA	5jtr_H	20	40	FC00218
P10997	SDA	5k5g_A	10	21	FC00217
P12493	SDA	2mgu_M	20	36	FC00205, FC00252
P14736	SDA	2m14_B	20	14	FC00256
P18683	SDA	1hji_B	20	14	FC00316
P19483	SDA	2jmx_B	30	22	FC00283
P19564	SDA	1biv_B	5	17	FC00322
P20366	SDA	2ks9_B	5	10	FC00272
P20366	SDA	2ksa_B	5	9	FC00272
P20366	SDA	2ksb_B	5	11	FC00272
P21698	SDA	2n0y_B	20	20	FC00246
P22770	SDA	1kc4_B	10	11	FC00310
P24565	SDA	1pnb_A	10	30	FC00304
P25024	SDA	1ilp_C	20	16	FC00313
P26554	SDA	2m3m_B	20	11	FC00254
P26715	SDA	2yu7_B	20	13	
P26729	SDA	1bon_B	10	22	FC00321
P29559	SDA	1wco_N	20	16	FC00299
P30989	SDA	2lyw_A	10	24	FC00257, FC00258
P30990	SDA	2lyw_B	10	13	FC00257, FC00258
P35228	SDA	5tp6_B	20	25	FC00197, FC00216
P37107	SDA	2hug_B	20	14	FC00285
P38862	SDA	2li5_B	19	31	FC00264
P48025	SDA	2lct_B	20	13	FC00152
P52907	SDA	1mq1_C	17	12	FC00158
P52907	SDA	1mq1_D	17	12	FC00158
P55284	SDA	2koh_B	20	12	FC00182
P55957	SDA	1zy3_B	10	20	FC00294
P58928	SDA	4b1q_P	20	6	FC00224
P61073	SDA	2k04_B	20	38	FC00277
P61073	SDA	2k04_D	20	37	FC00277
P61073	SDA	2k05_B	20	38	FC00277
P61073	SDA	2k05_D	20	38	FC00277
P61073	SDA	2n55_B	20	40	FC00277
P61830	SDA	2rnw_B	20	13	FC00233
P61830	SDA	2rnx_B	20	13	FC00233
P61830	SDA	2rsn_B	20	17	FC00234
P68249	SDA	2h3s_A	20	7	FC00287
P68249	SDA	2h3t_B	20	7	FC00287
P68249	SDA	2h4b_A	20	8	FC00287
P68249	SDA	2h4b_B	20	8	FC00287
P68431	SDA	6e83_A	20	8	FC00115

P68431	SDA	6e86_A	20	6	FC00115
P68431	SDA	2m0o_B	20	11	FC00116
P68431	SDA	2lgg_B	20	12	FC00117
P68431	SDA	4bd3_B	10	11	FC00118
P68433	SDA	1guw_B	25	18	FC00159
P84092	SDA	6rh6_B	30	15	FC00207
P84233	SDA	2l12_B	20	15	FC00206
Q07794	SDA	6f0y_B	10	14	FC00201
Q32PK0	SDA	2fci_B	16	13	FC00292
Q3BDD9	SDA	2l3r_B	15	11	FC00266
Q53971	SDA	1o9a_B	15	24	
Q61036	SDA	1ees_B	20	46	FC00132, FC00319
Q8CVI4	SDA	2vda_B	10	28	FC00228
Q8IVP5	SDA	2n9x_B	20	17	FC00239
Q8JJY9	SDA	2kqf_B	10	13	
Q92133	SDA	2l1b_B	20	15	FC00269
Q9BML6	SDA	5x9x_B	20	30	
Q9BML7	SDA	5x9x_A	20	29	
Q9R9T7	SDA	2jxh_A	30	34	FC00280
Q9R9T7	SDA	2jxh_B	30	34	FC00280

Uniprot: Uniprot identifier of the protein. **Dataset**: classification, D_exp: proteins, which were observed to drive formation of condensates, D_pred: proteins, which were predicted to drive condensate formation using the FuzDrop method, SDA:specific disordered assemblies. **PDB and chain**: PDB identifier of the complex structure and chain identifier for the disor-dered/droplet-driving partner. **Number of models**: the number of conformations deposited in the NMR ensemble. **Inter-face length**: the number of amino acid residues, which are in contact with the partner. **FuzDB**: Identifier in FuzDB, data- base of Fuzzy interactions (fuzdb.org [2]).

Reference

1. Hatos, A.; Hajdu-Soltesz, B.; Monzon, A.M.; Palopoli, N.; Alvarez, L.; Aykac-Fas, B.; Bassot, C.; Benitez, G.I.; Bevilacqua, M.; Chasapi, A.; et al. DisProt: Intrinsic protein disorder annotation in 2020. *Nucleic Acids Res.* **2020**, *48*, D269–D276. https://doi.org/10.1093/nar/gkz975.
2. Hatos, A.; Monzon, A.M.; Tosatto, S.C.E.; Piovesan, D.; Fuxreiter, M. FuzDB: A new phase in understanding fuzzy interactions. *Nucleic Acids Res.* **2021**. https://doi.org/10.1093/nar/gkab1060.
